# Dose–response relationship between functional pain interference and nonmedical analgesic use: Findings from a nationally representative Canadian survey

**DOI:** 10.1080/24740527.2018.1452147

**Published:** 2018-04-12

**Authors:** Pauline Voon, Jane A. Buxton, Evan Wood, Julio S. Montaner, Thomas Kerr

**Affiliations:** a British Columbia Centre for Excellence in HIV/AIDS, St. Paul’s Hospital, Vancouver, BC, Canada; b School of Population and Public Health, Faculty of Medicine, University of British Columbia, Vancouver, BC, Canada; c Harm Reduction Program, BC Centre for Disease Control, Vancouver, BC, Canada; d Department of Medicine, University of British Columbia, St. Paul’s Hospital, Vancouver, BC, Canada

**Keywords:** pain, addiction, nonmedical, substance abuse, misuse, adolescents, epidemiology, sex differences, opioids, population

## Abstract

**Background:**

Despite the epidemic of nonmedical analgesic use (NMAU) in North America, there is a scarcity of research quantifying the effect of pain on NMAU.

**Aims:**

This study sought to investigate the relationship between NMAU and functional pain interference, defined as the perceived level of interference in performing activities of daily living due to pain, in a population-based sample of the general Canadian population.

**Methods:**

Data from the 2012 Canadian Community Health Survey (CCHS)–Mental Health, a nationally representative cross-sectional survey, were used to conduct bivariable and multivariable logistic regression analyses.

**Results:**

The weighted prevalences of pain and NMAU were 20.6% and 6.6%, respectively. After adjusting for age, sex, education, culture/race, and chronic mental health diagnosis, a dose–response relationship was observed between higher functional pain interference and increased odds of NMAU, ranging from 1.61 (95% confidence interval [CI], 1.22–2.12) to 2.98 (95% CI, 2.21–4.01) from the lowest to the highest levels of functional pain interference. Elevated odds of NMAU were also observed among younger respondents aged 20–29 years and 15–19 years, respondents with a chronic mental illness diagnosis, and males. Secondary analyses revealed that the dose–response relationship between greater function pain interference and increased odds of NMAU persisted within subgroups with and without mental illness, as well as within subgroups aged 40 to 69.

**Conclusions:**

These findings highlight the potential role of pain on increasing NMAU and the need for targeted strategies to reduce harms of NMAU among high-risk subgroups such as young adults.

## RÉSUMÉ

**Contexte**: Malgré l’épidémie d’usage non médical d’analgésiques en Amérique du Nord, les études quantifiant l’effet de la douleur sur l’usage non médical d’analgésiques sont peu nombreuses.

**But**: Cette étude visait à étudier le lien entre l’usage non médical d’analgésiques et l’interférence de la douleur sur le plan fonctionnel, définie comme le niveau d’interférence perçu dans la réalisation des activités quotidiennes attribuable à la douleur, au sein d’un échantillon populationnel issu de la population canadienne en général.

**Méthodes**: Des données tirées de l’Enquête sur la santé dans les collectivités canadiennes– Santé mentale 2012, une enquête transversale représentative au niveau national, ont été utilisées pour mener des analyses de régression logistique bivariée et multivariée.

**Résultats**: La prévalence pondérée de la douleur et de l’usage non médical d’analgésiques était de 20,6 % et de 6,6 % respectivement. Après les ajustements liés à l’âge, au sexe, à l’éducation, à la culture ou à la race, ainsi qu’à un diagnostic de maladie mentale chronique, une relation dose - réponse a été observée entre un niveau plus élevé d’interférence de la douleur sur le plan du fonctionnement et l’augmentation des rapports de cotes de l’usage non médical d’analgésiques, qui allaientt de 1,61 (95 % IC: 1,22 – 2,12) à 2,98 (95 % IC : 2,21 – 4,01), du niveau le plus faible d’interférence de la douleur sur le plan du fonctionnementau au niveau le plus élevé. Des rapports de cote de l’usage non médical d’analgésiques élevés ont aussi été observés chez les plus jeunes répondants âgés de 20 à 29 ans et de 15 à 19 ans, ainsi que chez les répondants ayant reçu un diagnostic de maladie mentale chronique et chez les hommes. Des analyses secondaires ont révélé que la relation dose – réponse entre une plus grande interférence de la douleur sur le plan du fonctionnement et des rapports de cotes de l’usage non médical d’analgésiques plus élevés persistait au sein de sous-groupes atteints et non atteints de maladie mentale, ainsi qu’au sein de sous-groupes de participants âgés de 40 à 69 ans.

**Conclusions**: Ces résultats mettent en lumière le rôle potentiel de la douleur dans l’augmerntation de l’usage non médical d’analgésiques et la nécessité d’avoir recours à des stratégies ciblées pour réduire les effets néfastes causés par l’usage non médical d’analgésiques chez des sous-groupes à haut risque tel que les jeunes adultes.

## Introduction

Worldwide, pain is one of the most common reasons for seeking medical care, representing approximately 20% to 50% of primary care visits^1,2^ and up to 78% of hospital emergency department visits.^3^ In Canada, the prevalence of chronic pain is estimated to be 15% to 29%,^4^ which is greater than the national prevalence of diabetes, heart disease, and cancer combined.^5–7^ In recent years, efforts to improve pain management^8,9^ have contributed to increases in prescribing and dispensation of pain medications, particularly opioid analgesics.^10,11^ This rise in prescription analgesic distribution has coincided with an escalating epidemic of pharmaceutical diversion to street-based drug markets, misuse, dependence, and addiction.^12,13^ Correspondingly, the number of prescription analgesic-related overdoses and fatalities has risen at an alarming rate,^14,15^ with the number of deaths related to drug overdoses (primarily from opioid-based drugs, from either licit or illicit sources) surpassing the number of deaths from impaired motor vehicle accidents to become among the leading causes of injury-related death among adults in several North American settings.^16,17^

Though varying definitions exist, analgesic misuse or nonmedical analgesic use (NMAU) generally refers to the use of analgesics in any way other than directed by a prescription (e.g., alternate route, dose or frequency, or use of analgesics obtained from acquaintances or street-based drug markets).^18^ Previous studies have found significant positive correlations between pain and NMAU,^19,20^ as well as a high prevalence of co-occurring pain and NMAU.^21,22^ The frequency of concurrent pain and NMAU may be in part due to practitioners denying prescriptions for analgesia as a result of concerns regarding dependence, misuse, or diversion.^23,24^ Consequently, individuals may feel stigmatized and subsequently avoid seeking health care, opting instead to self-manage pain using diverted analgesics.^25,26^ Alternatively, individuals may transition to NMAU secondary to developing tolerance or addiction to prescribed analgesics and potentially even transition to heroin or other illicit opioid use.^27–30^

Despite these growing public health concerns, there is a scarcity of research investigating dose–effect relationships between pain and NMAU, particularly in the context of confounding factors such as age and mental illness. Therefore, to address this research gap and inform public health approaches to addressing concurrent pain and prescription analgesia misuse, the current study utilizes data from a national health survey to investigate the association between pain and NMAU in Canada.

## Methods

### Setting

Data for these analyses were derived from the Public Use Microdata File of the Canadian Community Health Survey (CCHS)–Mental Health, a national cross-sectional survey conducted by Statistics Canada. Between January and December 2012, data were collected from a nationally representative sample of individuals aged 15 years and older. The sample represents approximately 97% of the population that lives in the ten Canadian provinces, and the 3% excluded from the sample includes persons living in the three territories, persons living on Aboriginal reserves or settlements, persons who are full-time members of the Canadian Forces, and persons who are institutionalized. The CCHS–Mental Health used a multistage stratified cluster design to attain the sample of respondents. In general, each Canadian province is divided into regions based on population size. Within each region, homogenous strata are created by geographic or socioeconomic characteristics. Within each stratum, individual households are grouped into clusters. Independent samples of clusters are randomly selected using probability proportional to size. Finally, individual households are systematically sampled from each randomly selected cluster, with one randomly selected respondent permitted per household. In total, 25,113 interviews were conducted using computer assisted personal interviewing, which yielded an overall person-level response rate of 86.3%. The sampling and interview methods of the CCHS–Mental Health are described in detail by Statistics Canada.^31^

### Sample

The present analysis included all respondents of the CCHS–Mental Health who provided valid responses to the outcome measure of nonmedical analgesic use, the explanatory variable of functional pain interference, and the known confounders (listed below). Respondents who provided invalid responses (e.g., “don’t know,” “refused,” or “not stated”) for any of the study variables were excluded.

### Primary measurements and outcomes

The outcome of interest for the present analysis was a lifetime history of ever using nonmedical analgesics. The definition of “nonmedical” use described by Statistics Canada to survey respondents included use without the recommendation of a health professional, use in greater amounts than directed by a health professional, or use for any reason other than directed by a health professional. Nonmedical analgesic use was coded dichotomously by Statistics Canada as a response of “yes” to either of the questions: “Have you ever used a pain killer nonmedically?” or “Was your use ever so regular that you felt that you could not stop using the pain killer prescribed to you?” versus a response of “no” to both of these questions. Examples of analgesics described to respondents during the interview included codeine, morphine, and oxycodone. Over-the-counter analgesics were also included if they were used nonmedically. Similar measures of NMAU have been used in other national surveys and have been found to be valid and reliable.^32^

The explanatory variable of interest was pain functional interference, which was derived by Statistics Canada based on respondents’ perceived “usual” levels of interference in performing activities of daily living due to pain. Respondents were asked about their pain interference prior to the questions on NMAU. Responses were categorized as “no pain or discomfort,” “pain prevents no activities,” “pain prevents a few activities,” “pain prevents some activities,” or “pain prevents most activities.”

Potentially confounding variables, which were included based on their demonstrated associations with pain and NMAU in past research,^33^ included age (10-year age groupings, with the exception of the youngest age group of 15–19 years), sex (female versus male), highest level of education completed by the respondent (less than versus greater or equal to secondary school graduation), culture/race (white versus non-white), and chronic mental health diagnosis (yes to any of chronic fatigue, mood disorder including depression, anxiety disorder, posttraumatic stress disorder, or attention deficit disorder, versus no to all).

### Study design

All analyses were weighted using sampling weights provided by Statistics Canada to adjust for uneven probabilities of selection due to the nonrandom sampling scheme, in order to generate population-based estimates. Next, univariable descriptive statistics were generated to investigate the characteristics of the sample. Bivariable analyses were conducted to obtain unadjusted odds ratios, 95% confidence intervals, and *P* values for the relationships between the explanatory, confounding, and outcome variables. Finally, a multivariable logistic regression model was constructed to investigate the association between the explanatory and outcome variables, while adjusting for the confounding varibles. Inclusion and exclusion of variables in the model were tested to determine impact on parameter estimates, and comparisons of Akaike information criterion were used to assess goodness of fit. All *P* values were two-sided. Significant associations were defined as *P *< 0.05. As a secondary analysis, the dose–effect relationship between increasing levels of functional pain interference on the odds of NMAU were examined within strata of chronic mental illness and age, while controlling for confounders found to be relevant from the final multivariable model. All analyses were conducted using SAS software version 9.3 (SAS Institute Inc., Cary, NC). The present study adheres to standards regarding data privacy, confidentiality, and use of publicly available data as set out by the Canadian Statistics Act^34^ and the University of British Columbia Policy on Research and Other Studies Involving Human Subjects, Item 1.3.1.^35^

## Results

**Figure 1.**
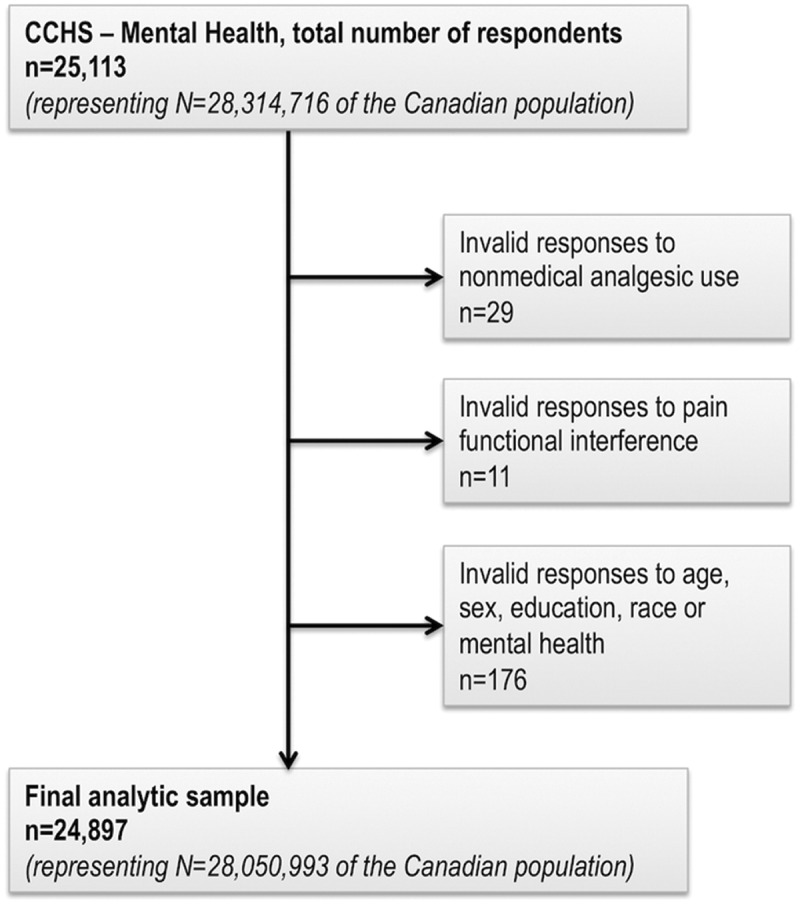
Study sample derived from the Canadian Community Health Survey–Mental Health.

The final analytic sample (*n* = 24,897) after exclusion of respondents with missing data (*n* = 216; 0.86% of the original sample) is described in Figure 1 and Table 1. The sample was fairly evenly distributed across the age groups, with less representation in the youngest and oldest age groupings. Sex was distributed nearly equally (49.2% male, 50.8% female). Approximately one in five respondents reported being non-white (23.0%) or having completed less than secondary school graduation (18.1%). A chronic mental illness diagnosis was reported by 12.2% of respondents.

**Table 1.** Weighted estimates of functional pain interference from Canadian Community Health Survey–Mental Health (2012).^a^

Variable	Total ’000 (%)	No pain ’000 (%)	Pain prevents no activities ’000 (%)	Pain prevents few activities ’000 (%)	Pain prevents some activities ’000 (%)	Pain prevents most activities ’000 (%)
Functional pain interference	28 051 (100.0)	22 261 (79.4)	1733 (6.2)	1725 (6.1)	1314 (4.7)	1018 (3.6)
Nonmedical analgesic use						
No	26 193 (93.4)	21 085 (75.2)	1594 (5.7)	1547 (5.5)	1116 (4.0)	850 (3.0)
Yes	1858 (6.6)	1176 (4.2)	139 (0.5)	177 (0.6)	198 (0.7)	168 (0.6)
Age						
15–19 years	2284 (8.1)	20 995 (7.5)	56 (0.2)	79 (0.3)	30 (0.1)	19 (0.1)
20–29 years	4414 (15.7)	38 636 (13.8)	184 (0.7)	180 (0.6)	113 (0.4)	74 (0.3)
30–39 years	4418 (15.7)	38 131 (13.6)	208 (0.7)	180 (0.6)	124 (0.4)	94 (0.3)
40–49 years	4901 (17.5)	38 891 (13.9)	282 (1.0)	332 (1.2)	204 (0.7)	194 (0.7)
50–59 years	5208 (18.6)	38 823 (13.8)	400 (1.4)	333 (1.2)	349 (1.2)	243 (0.9)
60–69 years	3682 (13.1)	26 230 (9.4)	301 (1.1)	314 (1.1)	241 (0.9)	203 (0.7)
70–79 years	2109 (7.5)	13 994 (5.0)	201 (0.7)	210 (0.7)	180 (0.6)	119 (0.4)
≥80 years	1035 (3.7)	6907 (2.5)	101 (0.4)	97 (0.3)	73 (0.3)	73 (0.3)
Sex						
Male	13 800 (49.2)	11 303 (40.3)	833 (3.0)	708 (2.5)	525 (1.9)	431 (1.5)
Female	14 251 (50.8)	10 958 (39.1)	900 (3.2)	1017 (3.6)	789 (2.8)	587 (2.1)
Highest education completed						
<Secondary school graduation	5066 (18.1)	3715 (13.2)	368 (1.3)	382 (1.4)	305 (1.1)	296 (1.1)
≥Secondary school graduation	22 985 (81.9)	18 545 (66.1)	1366 (4.9)	1343 (4.8)	1009 (3.6)	722 (2.6)
Culture/race						
White	21 598 (77.0)	16 931 (60.4)	1390 (5.0)	1408 (5.0)	1053 (3.8)	816 (2.9)
Non-white	6453 (23.0)	5329 (19.0)	343 (1.2)	317 (1.1)	261 (0.9)	202 (0.7)
Chronic mental illness^b^						
No	24 621 (87.8)	20 229 (72.1)	1514 (5.4)	1323 (4.7)	936 (3.3)	619 (2.2)
Yes	3430 (12.2)	2032 (7.2)	220 (0.8)	402 (1.4)	378 (1.3)	399 (1.4)

^a^All frequencies and percentages are probability weighted using Statistics Canada sampling weights. Frequencies displayed in this table have been rounded to the nearest thousand. Percentages may not total to 100% due to rounding.

^b^Defined as “yes” to any of chronic fatigue, mood disorder including depression, anxiety disorder, posttraumatic stress disorder, or attention deficit disorder, versus “no” to all

In total, 6.6% of the sample reported a lifetime history of NMAU, representing approximately 1.8 million Canadians. The majority of the sample reported no pain (79.4%). Among the remaining respondents with pain (20.6%), there was a gradual decrease in the proportion reporting pain that prevented no activities (6.2%) to the percentage reporting pain that prevented most activities (3.6%). Increasing proportions of NMAU were observed within each increasing level of functional pain interference (e.g., NMAU was reported by 5.4% of respondents with no pain, compared to 14.5% of respondents with the highest level of functional pain interference). In terms of the confounding variables, the prevalence of NMAU was higher among males, respondents who had not graduated from secondary school, respondents who were aged 20–29 and 50–59 years, and respondents who identified as white.

**Figure 2.**
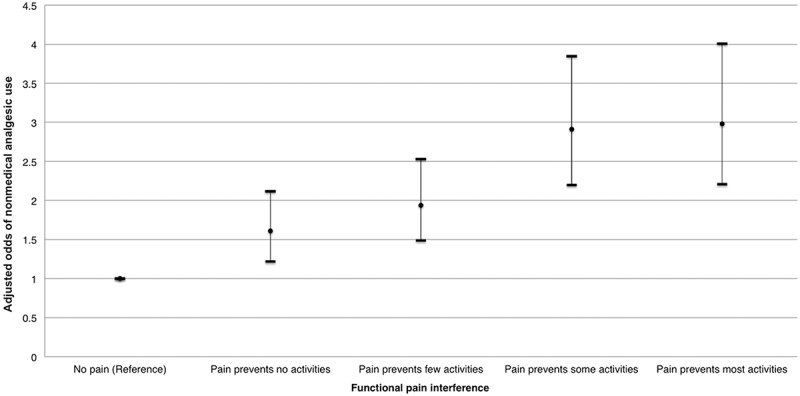
Adjusted odds ratios and 95% confidence intervals for nonmedical analgesic use stratified by functional pain interference, controlling for age, sex, highest education completed, culture/race, and chronic mental illness.

Table 2 presents the bivariable and multivariable logistic regression analyses. Evidence of a dose–response relationship was observed between higher functional pain interference and increased odds of lifetime NMAU, which remained after adjusting for age, sex, education, race, and mental illness (Figure 2). Specifically, compared to respondents with no pain, the adjusted odds of NMAU increased with each increasing level of functional pain interference. The dose–response effect is further illustrated by the relatively narrow 95% CIs that exclude the null across pain levels.

**Table 2.** Bivariable and multivariable weighted^a^ logistic regression examining the relationship between functional pain interference and NMAU.

Variable	NMAU OR	
	Unadjusted OR (95% CI)	Adjusted OR^b^ (95% CI)
Functional pain interference		
No pain	Reference	Reference
Pain prevents no activities	1.57 (1.19–2.05)	1.61 (1.22–2.12)
Pain prevents a few activities	2.06 (1.59–2.66)	1.94 (1.49–2.53)
Pain prevents some activities	3.18 (2.43–4.18)	2.91 (2.20–3.85)
Pain prevents most activities	3.54 (2.67–4.70)	2.98 (2.21–4.01)
Age		
15–19 years	1.78 (1.17–2.70)	2.11 (1.38–3.24)
20–29 years	2.19 (1.51–3.17)	2.47 (1.67–3.66)
30–39 years	1.38 (0.96–1.97)	1.56 (1.06–2.28)
40–49 years	1.62 (1.11–2.37)	1.67 (1.13–2.47)
50–59 years	1.90 (1.27–2.84)	1.82 (1.20–2.74)
60–69 years	1.56 (1.05–2.34)	1.50 (0.99–2.27)
70–79 years	1.25 (0.84–1.85)	1.18 (0.79–1.77)
≥80 years	Reference	Reference
Sex		
Male	1.20 (1.01–1.44)	1.32 (1.10–1.58)
Female	Reference	Reference
Highest education completed		
<Secondary school graduation	1.09 (0.89–1.34)	1.03 (0.83–1.28)
≥Secondary school graduation	Reference	Reference
Culture/race		
White	Reference	Reference
Non-white	0.79 (0.63–0.99)	0.83 (0.66–1.04)
Chronic mental illness^c^		
No	Reference	Reference
Yes	3.09 (2.57–3.72)	2.39 (1.98–2.87)

^a^All estimates are probability weighted using Statistics Canada sampling weights.

^b^Adjusted for age, sex, highest education completed, culture/race, and chronic mental illness.

^c^Defined as “yes” to any of chronic fatigue, mood disorder including depression, anxiety disorder, post traumatic stress disorder, or attention deficit disorder, versus “no” to all.

NMAU = nonmedical analgesic use; OR = odds ratio; CI = confidence interval.

**Figure 3.**
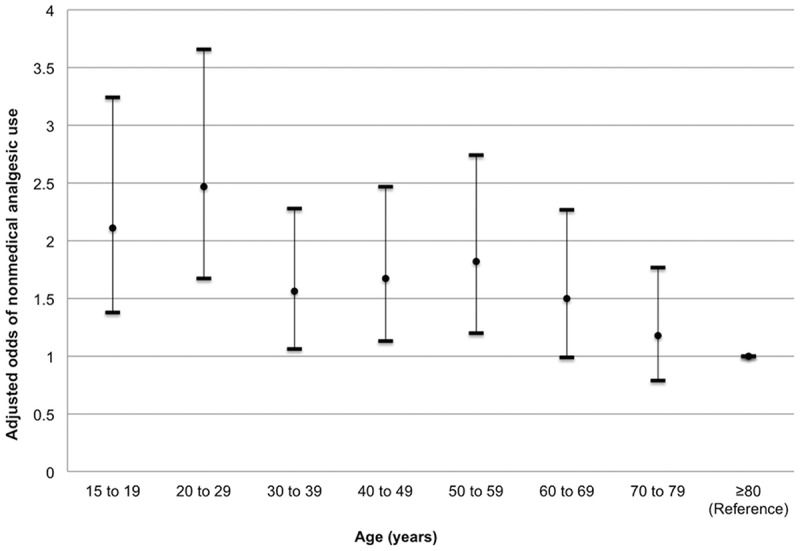
Adjusted odds ratios and 95% confidence intervals for nonmedical analgesic use stratified by age, controlling for functional pain interference, sex, highest education completed, culture/race, and chronic mental illness.

In terms of the potentially confounding variables, the odds of NMAU was higher among males (adjusted odds ratio [AOR] = 1.32, 95% CI, 1.10–1.58) and respondents with a chronic mental illness diagnosis (AOR = 2.39, 95% CI, 1.98–2.87). Mental illness had the strongest confounding effect that attenuated the main effects by 10% to 28% across the different pain categories. For age, the greatest odds of NMAU were observed among younger respondents who were 20 to 29 years (AOR = 2.47, 95% CI, 1.67–3.66) and 15 to 19 years (AOR = 2.11, 95% CI, 1.38–3.24; Figure 3). Respondents reporting non-white culture/race had a decreased odds of NMAU (nonsignificant in adjusted analysis), and education was not significantly associated with NMAU. There was sufficient power for investigating the association between each stratum of pain and NMAU (all power calculations performed at a two-sided significance level of 0.05 resulted in power of 0.99 or greater to detect a minimal difference of 3.3% in the proportion of NMAU among those whose pain prevented no activities versus those who reported having no pain).

Table 3 presents the secondary analysis showing that the dose–response relationship between higher functional pain interference and increased odds of NMAU generally persisted within strata of individual who did and did not have mental illness. Within strata of age groupings, this dose–response relationship was also observed among adults from age 40 to 69. However, the dose–response effect was not as evident in the youngest and oldest subgroups, in which wider confidence intervals were observed.

**Table 3.** Weighted^a^ multivariable logistic regression analyses examining the odds of functional pain interference on nonmedical analgesic use, within strata of chronic mental illness and age groupings.

Variable	Pain prevents no activities^b^ AOR (95% CI)	Pain prevents few activities^b^ AOR (95% CI)	Pain prevents some activities^b^ AOR (95% CI)	Pain prevents most activities^b^ AOR (95% CI)
Chronic mental illness^c^				
No	1.41 (1.02–1.96)	2.06 (1.49–2.85)	2.67 (1.88–3.81)	2.75 (1.81–4.17)
Yes	2.30 (1.37–3.88)	1.70 (1.10–2.65)	3.43 (2.19–5.35)	3.37 (2.17–5.23)
Age^d^				
15–19 years	2.16 (0.66–7.02)	1.18 (0.49–2.83)	0.46 (0.13–1.70)	4.28 (1.00–18.21)
20–29 years	2.97 (1.65–5.35)	2.69 (1.34–5.38)	4.38 (2.00–9.60)	1.03 (0.42–2.56)
30–39 years	2.00 (1.05–3.79)	1.19 (0.59–2.40)	4.80 (2.38–9.67)	3.12 (1.44–6.77)
40–49 years	1.88 (0.94–3.76)	2.11 (1.08–4.10)	3.16 (1.65–6.06)	3.29 (1.57–6.86)
50–59 years	1.01 (0.50–2.03)	1.77 (1.03–3.06)	2.15 (1.23–3.74)	2.80 (1.65–4.75)
60–69 years	0.67 (0.32–1.38)	1.19 (0.61–2.32)	3.14 (1.64–6.04)	4.07 (2.02–8.21)
70–79 years	1.34 (0.60–3.01)	3.24 (1.65–6.37)	1.75 (0.74–4.16)	2.77 (1.19–6.46)
≥80 years	3.68 (1.42–9.55)	2.57 (1.07–6.22)	3.37 (1.38–8.24)	3.66 (1.42–9.45)

^a^All estimates are probability weighted using Statistics Canada sampling weights.

^b^Compared to reference category of “no pain.”

^c^Adjusted for sex, highest education completed, culture/race, and age.

^d^Adjusted for sex, highest education completed, culture/race, and chronic mental illness.

AOR = adjusted odds ratio; CI = confidence interval.

## Discussion

The present study is the first to demonstrate an independent dose–response relationship between higher reported functional pain interference and increased odds of NMAU in a large, nationally representative sample of the Canadian population. This dose–response relationship persisted within strata of chronic mental illness and adults aged 40 to 69.

The prevalence of lifetime NMAU in this study (6.6%) is similar to the prevalence of NMAU reported from the U.S. National Survey on Drug Use and Health (4.9%) and from the Centre for Addiction and Mental Health Monitor of the Ontario general population (7.7%).^36,37^ Put into context, the lifetime prevalence of NMAU in this study is greater than the lifetime prevalence of methamphetamine, ecstasy, and heroin use combined in the general Canadian population (5.6%),^38^ which illustrates the scale of this public health problem. Furthermore, the pooled prevalence of pain in this study is consistent with the estimated prevalence of chronic pain in Canada (i.e., approximately 20%).^39^ Similarly, a higher prevalence of pain was found among individuals reporting NMAU in this study (36.7%), which is comparable to other studies that have found higher rates of pain among individuals who use analgesics nonmedically in North America.^40^ Given that the majority of these population-level estimates are based on U.S. data, this study adds evidence on the pervasiveness of pain and NMAU from the Canadian context.

The positive independent association between higher pain interference and increased odds of NMAU observed in this study is generally consistent with other research. In the only other study of this relationship in a nationally representative sample, higher levels of pain were positively and independently associated with increased likelihood of past-year NMAU among respondents to the U.S. National Epidemiology Survey on Alcohol and Related Conditions.^41^ Similarly, in studies of state-level and hospital-level samples, higher levels of functional pain interference were correlated with significantly greater odds of prescription drug use disorder, high-risk analgesic misuse, and patient perception of analgesic-related problems or concerns.^42–44^ The correlation between pain and NMAU may be explained by a variety of factors that may be difficult to disentangle, such as self-medication for pain secondary to withheld or noneffective analgesic prescriptions for managing pain; self-medication for dependence or withdrawal, potentially related to iatrogenic addiction from prescribed analgesics for an initial pain concern; or deliberate misuse for the purpose of euphoria in individuals with or without concurrent pain.^33,45^ However, some studies have not found associations between pain and NMAU,^46,47^ which may be explained by varying sample restrictions or discrepancies in the definitions of pain or NMAU used across studies (e.g., measures based on pain severity scales, urine test results, or diagnostic codes for pain or opioid dependence/abuse). This study adds evidence of a dose–response effect between pain and NMAU that remained apparent in subgroups with and without mental illness, which may suggest that although mental illness may be a significant predictor of NMAU, the influence of pain on NMAU may be independent of it.

Furthermore, the present findings highlight the significantly increased odds of NMAU among young adults, which is counterintuitive given than older adults would be hypothesized to have greater functional pain interference and greater access to prescription analgesia. In subanalyses within age strata, the dose–response effect between greater pain and increased odds of NMAU persisted in adults aged 40 to 69; however, in the youngest and oldest subgroups, the dose–response effect was not as evident and wider confidence intervals were observed. This may suggest that there is greater variability in pain or NMAU in the youngest and oldest age groups. Therefore, further research and prevention efforts are needed to address the methods and motives for NMAU among young adults in Canada, including the potential role of increased access to diverted prescription analgesics for this population.^48,49^

The present study has certain limitations. First, the use of self-reported data to ascertain NMAU is susceptible to socially desirable reporting bias that may lead to underreporting,^37,50^ which would likely contribute to underestimation of the prevalence of NMAU and nondifferential bias of effect sizes toward the null. As a result, the estimates in the present study are likely to be conservative. Additionally, temporality and causal associations cannot be ascertained from this study, particularly given that a lifetime measure of NMAU was used. However, as previously noted, the positive independent association between pain and NMAU in this study appears to be consistent with past research using past-year NMAU measures. Furthermore, important details such as acute versus chronic pain, nonmedical use of opioid analgesics versus less high-risk analgesics (e.g., over-the-counter medications), the intensity of or motives for NMAU (e.g., use for undertreated pain versus recreational use), or source of analgesics (e.g., use of one’s own prescription versus medication obtained from another person) were not captured in the present study. Therefore, more detailed data collection is needed in future research to fully understand the patterns underlying concurrent pain and NMAU.^51,52^ Finally, though the use of sampling weights adjusts for representativeness, bootstrapped weights were not used to correct for the impact of the clustered sampling design on variance estimates, because such weights are not available for use with the CCHS Public Use Microdata File. As such, all variance estimates in this study are approximate. Despite these limitations, the present study provides a nationally representative, well-powered analysis revealing an independent dose–response relationship between pain and NMAU.

Given the severity of overdose fatalities and related harms stemming from NMAU in North America, clinical and policy interventions have thus far centered on restricting the supply and distribution of high-risk analgesics through efforts to reduce and monitor physician prescribing of opioid analgesics.^12^ However, strategies to promote appropriate pain management are often overlooked in these interventions, which emphasize preventing analgesic abuse and diversion without adequately addressing the potential root cause of pain.^53^ Given that the present study adds to the growing body of evidence suggesting that pain may play a significant role in NMAU, future efforts should be dedicated to research, education, and policies supporting safer, more effective alternatives to high-risk prescription analgesics for managing pain. Furthermore, this study highlights particular subgroups (i.e., young adults, males, and individuals with chronic mental illness) that may be at higher risk of NMAU and that may therefore benefit from targeted public health interventions. Ultimately, improved research and strategies to address pain and NMAU are urgently required to prevent the severe harms associated with this national epidemic.
